# Giant retroperitoneal lipoma in 62-year-old male: a rare benign tumor in an unusual location

**DOI:** 10.1093/jscr/rjaf859

**Published:** 2025-10-26

**Authors:** Abdulrahman Mohammed Abdulrahman Abouh, Mohammedbabalrahma Bashier Ahmed Koko, Esra Altayeb Alhady Massry, Ahmed Idris Abdelrahman Idris, Al-Siddig Mohammed Abdulrahman Abouh, Ahmed Abdalla Agab Eldour

**Affiliations:** Department of Surgery, El Obeid Teaching Hospital, El Obeid, North Kordofan, Sudan; Department of Surgery, El Obeid Teaching Hospital, El Obeid, North Kordofan, Sudan; Department of Surgery, El Obeid Teaching Hospital, El Obeid, North Kordofan, Sudan; Department of Surgery, El Obeid Teaching Hospital, El Obeid, North Kordofan, Sudan; Faculty of Medicine, University of Kordofan, El Obeid, North Kordofan, Sudan; Department of Pathology, Faculty of Medicine and Health Sciences, University of Kordofan, El Obeid, North Kordofan, Sudan

**Keywords:** retroperitoneal lipoma, liposarcoma, abdominal surgery, retroperitoneal tumors, case report

## Abstract

Lipomas occur elsewhere in the body; however, retroperitoneal lipomas are extremely rare, benign tumors. Unlike liposarcoma, it lacks malignant potential but may grow to a large size because of its spacious retroperitoneal cavity. We report a 62-year-old male presented with progressive abdominal distension for over 2 months. Imaging revealed a large retroperitoneal fatty mass (15.5 × 12.3 × 22.8 cm) herniating through the obturator and sciatic foramina, displacing pelvic organs. Laparotomy with complete excision was performed, and histopathology confirmed a benign lipoma. The patient was discharged without adverse events. Retroperitoneal lipomas are rare and often indistinguishable from liposarcomas on imaging. Histopathology remains crucial for diagnosis, and complete surgical excision provides excellent prognosis, as demonstrated in this case. Differential diagnosis of retroperitoneal masses should include retroperitoneal lipomas, although they are rare. Imaging is useful; however, histopathological examination is essential for definitive diagnosis.

## Introduction

There are many types of soft-tissue tumors, among which lipomas are the most common, arising from the benign proliferation of mature adipocytes. These lesions, which develop from mesenchymal cells, can appear in almost any site of the body. However, occurrence within the retroperitoneum is extremely uncommon, making such tumors rarely encountered in surgical practice [1].

Most lipomas are located in the subcutaneous tissue of the trunk or extremities, with retroperitoneal localization representing an unusual finding [2]. The main clinical difficulty is differentiating a simple retroperitoneal lipoma from a well-differentiated liposarcoma, since they may present with overlapping radiological features. Although cross-sectional imaging with computed tomography (CT) or magnetic resonance imaging (MRI) and, in some cases, CT-guided biopsy can provide valuable information, distinguishing between the two entities prior to surgery remains problematic [3].

In general, benign retroperitoneal tumors are well-defined and encapsulated, whereas malignant retroperitoneal tumors are much less common overall, accounting for <1% of all body neoplasms [4]. We present a rare case of retroperitoneal lipoma with extension through both the obturator and sciatic foramina into the groin and buttock, a presentation not previously reported. This case is described in line with the SCARE 2025 guidelines [5].

## Case presentation

**Figure 1:**
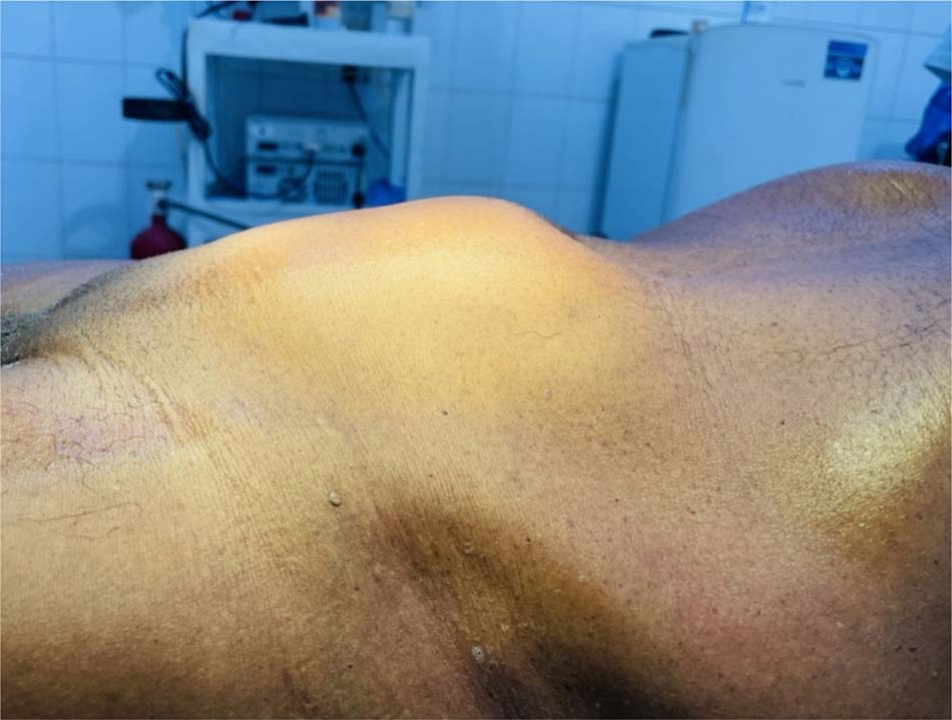
Preoperative clinical photograph of the patient showing visible suprapubic abdominal swelling extending up to the umbilicus (arrow).

During the course of 2 months, a 62-year-old male presented with abdominal pain. He reported abdominal distension in the last 2 weeks. He was afebrile, not pale or jaundiced, with a BP of 120/80 mmHg, pulse 88 bpm, RR 18 cpm, and distension in the suprapubic area (Fig. 1). Organomegally was not observed, and laboratory tests revealed the following finding:

- Normal full blood count
- Normal renal function and electrolytes
- Normal liver function test
- Normal random blood Glucose
- Urinalysis: pus cells

**Figure 2:**
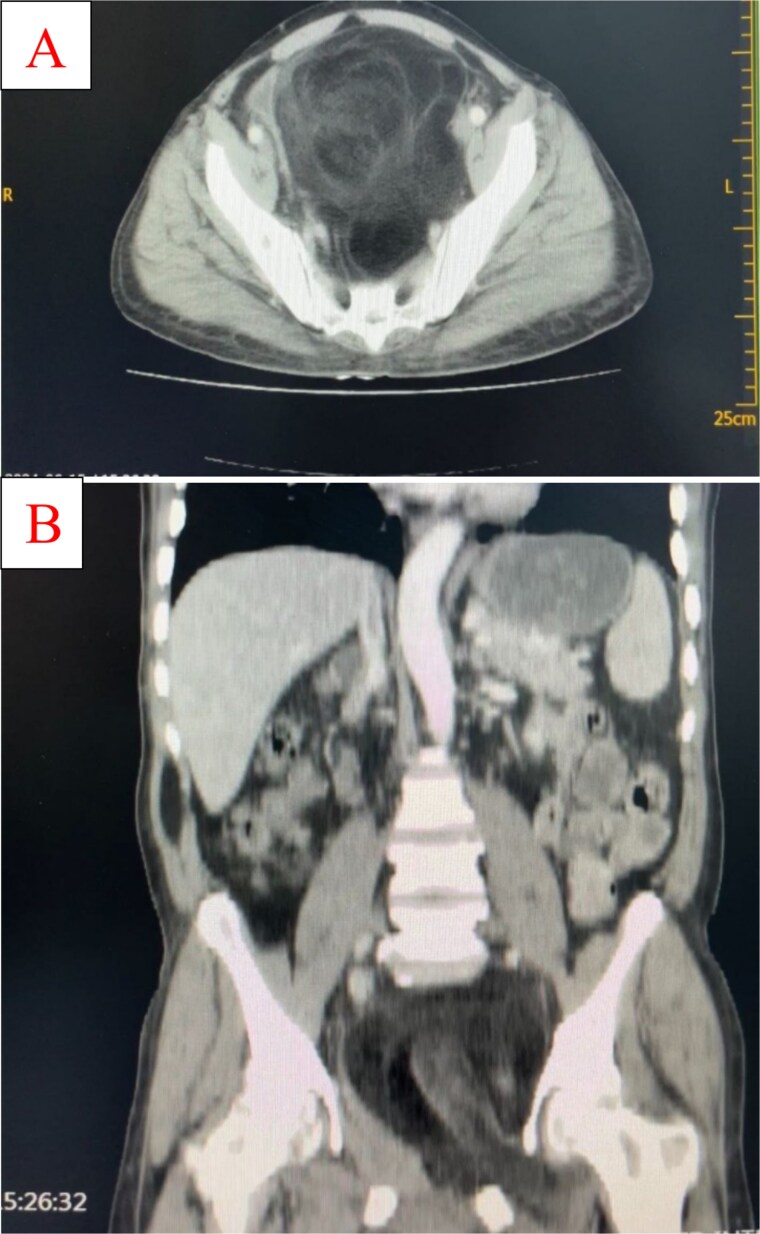
Coronal (A) and sagittal (B) CT images demonstrating a large retroperitoneal fatty mass with central soft tissue density. The mass extends through the left obturator and right greater sciatic foramina, displacing the bladder anteriorly and pelvic organs laterally.

Ultrasonography revealed a well-differentiated retroperitoneal mass measuring approximately 14 × 12 × 21 cm. The chest radiography and electrocardiography findings were normal. CT scan showed huge pelvi-abdominal fatty mass exhibiting central soft tissue component measuring 15.5 × 12.3 × 22.8 cm in the greatest anterioposterior, transverse, and coroncodal dimensions, respectively, and a mean density of −83 HU. The mass was well-defined; it is abutting the left pelvis side wall exiting (herniating) through the left obturator and the right greater sciatic foramina into the left groin and right buttock. The mass is compressing and displacing the bladder anteriorly and to the right side and the rectum and sigmoid to the right side against the pelvis side wall. The line of cleavage is preserved; no aggressive behavior or bone destruction (Fig. 2).

**Figure 3:**
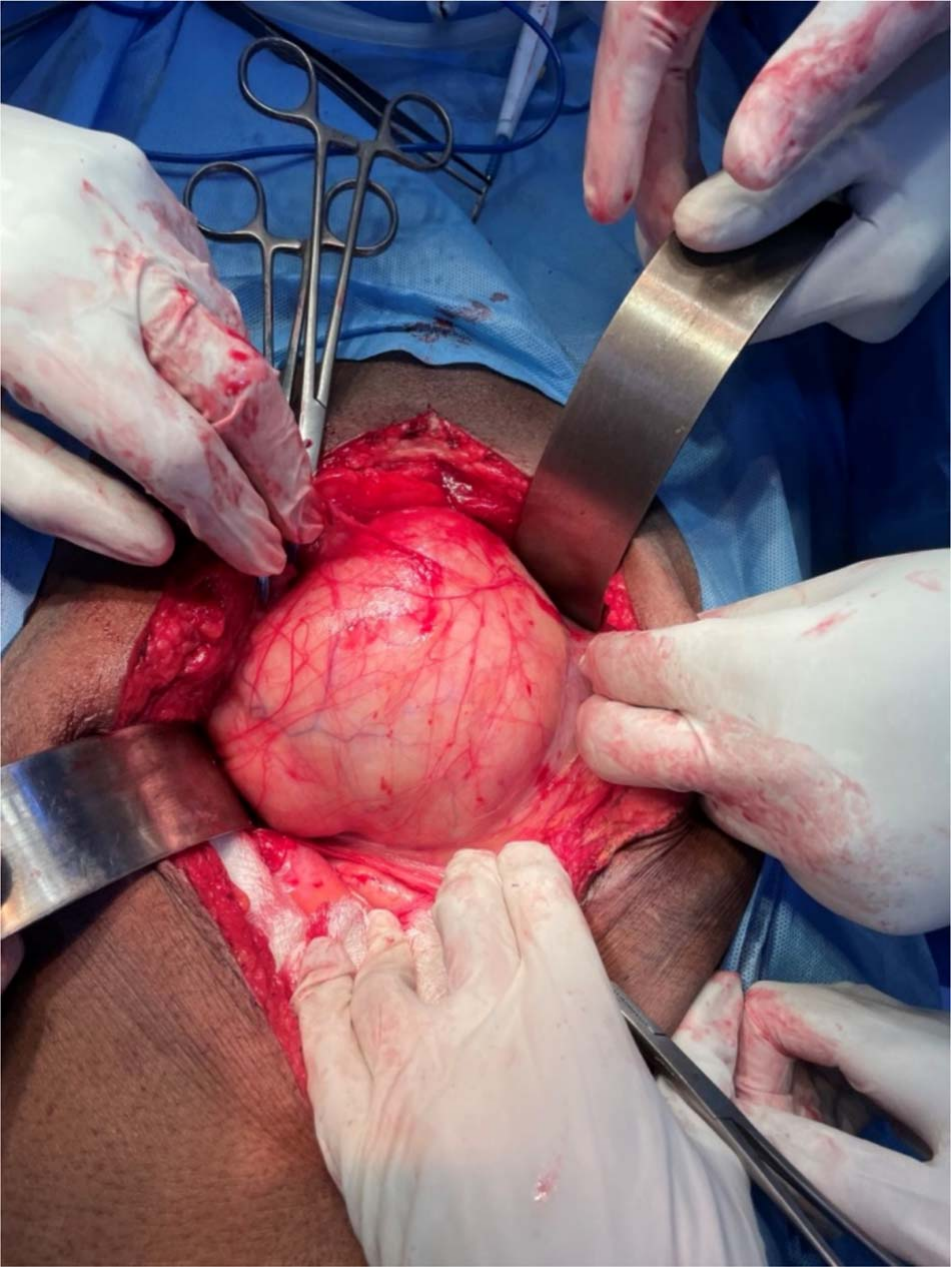
Intraoperative photograph showing the well-encapsulated giant retroperitoneal lipoma being mobilized during laparotomy.

A provisional diagnosis of a retroperitoneal lipoma was made. The patient underwent exploratory laparotomy via a midline incision. Intraoperatively, a giant, well-encapsulated retroperitoneal lipomatous mass was identified (Fig. 3), extending through both the left obturator foramen and right greater sciatic foramen. The tumor was carefully mobilized from the adjacent pelvic organs and the bladder was displaced anteriorly. Complete excision was achieved en bloc without injury to surrounding structures. Estimated blood loss was minimal, and no intraoperative complications occurred. Postoperatively, the patient recovered uneventfully, resumed oral intake within 24 h, and was discharged on the third postoperative day in good condition. At 3-month follow-up, he remained asymptomatic with no evidence of recurrence on clinical examination. Histopathology confirmed a benign lipoma.

## Discussion

Retroperitoneal lipomas are rare benign mesenchymal tumors, particularly in adults, with only isolated cases documented in the literature [4, 6–8]. Within the retroperitoneum, liposarcomas occur far more frequently than lipomas, making benign fatty tumors in this location exceptional and often difficult to diagnose preoperatively [6, 9].

Because the retroperitoneal cavity allows progressive but silent enlargement, these tumors often remain asymptomatic until they become very large. Patients may then present with non-specific complaints, such as abdominal swelling, discomfort, urinary symptoms, or pressure effects on adjacent viscera [8]. Some cases have been reported with extension into the inguinal or femoral canal, simulating hernias [7], or with pelvic or sciatic manifestations [10]. In the present case, progressive abdominal distension was the main symptom, consistent with prior reports [8].

Radiologically, retroperitoneal lipomas and well-differentiated liposarcomas appear similar, often manifesting as large, encapsulated fatty lesions with fine internal septations on CT or MRI [8]. Suspicion for liposarcoma increases when irregular septa, nodularity, or solid components are seen; however, imaging alone cannot confirm the diagnosis [9]. Histopathological examination is therefore essential. Unlike liposarcoma, lipomas contain mature adipocytes without atypia or lipoblasts, and lack molecular alterations such as MDM2 or CDK4 amplification [4, 9].

Surgical resection remains the definitive treatment, both for excluding malignancy and for relieving symptoms [4, 6, 8]. Complete removal with negative margins is recommended. Depending on tumor extent, various approaches have been described, including transgluteal [10], abdominoinguinal [4], or conventional open procedures [8]. For giant retroperitoneal tumors, open en bloc resection is generally the safest and most effective option. Prognosis following complete excision is excellent, with a very low risk of recurrence, in contrast to liposarcomas, which frequently recur locally even after resection [6, 8]. Nonetheless, periodic follow-up with imaging is prudent to rule out recurrence or misdiagnosis [4, 9].

This case adds to the limited body of literature on adult retroperitoneal lipomas and underscores the diagnostic challenge in distinguishing them from liposarcomas. It also highlights the importance of histopathology in confirmation and complete surgical excision as curative treatment, thereby contributing to greater clinical awareness and more informed surgical decision-making.

In conclusion, retroperitoneal lipomas are rare, often reaching large dimensions due to the expansile nature of the retroperitoneal cavity. They present a diagnostic challenge, especially in differentiating them from liposarcomas. Our case, with dual herniation through both the obturator and sciatic foramina, underscores the importance of thorough imaging, careful surgical planning, and histopathological confirmation. Complete excision is curative, and reporting such unusual cases expands awareness and assists in clinical decision-making.
